# Expanding the mammalian phenotype ontology to support automated exchange of high throughput mouse phenotyping data generated by large-scale mouse knockout screens

**DOI:** 10.1186/s13326-015-0009-1

**Published:** 2015-03-25

**Authors:** Cynthia L Smith, Janan T Eppig

**Affiliations:** Mouse Genome Informatics, The Jackson Laboratory, Bar Harbor, ME 04609 USA

**Keywords:** Phenotype, Ontology, Mouse, Data integration, Database

## Abstract

**Background:**

A vast array of data is about to emerge from the large scale high-throughput mouse knockout phenotyping projects worldwide. It is critical that this information is captured in a standardized manner, made accessible, and is fully integrated with other phenotype data sets for comprehensive querying and analysis across all phenotype data types. The volume of data generated by the high-throughput phenotyping screens is expected to grow exponentially, thus, automated methods and standards to exchange phenotype data are required.

**Results:**

The IMPC (International Mouse Phenotyping Consortium) is using the Mammalian Phenotype (MP) ontology in the automated annotation of phenodeviant data from high throughput phenotyping screens. 287 new term additions with additional hierarchy revisions were made in multiple branches of the MP ontology to accurately describe the results generated by these high throughput screens.

**Conclusions:**

Because these large scale phenotyping data sets will be reported using the MP as the common data standard for annotation and data exchange, automated importation of these data to MGI (Mouse Genome Informatics) and other resources is possible without curatorial effort. Maximum biomedical value of these mutant mice will come from integrating primary high-throughput phenotyping data with secondary, comprehensive phenotypic analyses combined with published phenotype details on these and related mutants at MGI and other resources.

## Background

The accessibility of the mouse genome to genetic manipulation, biochemical and molecular experimentation, and the availability of its full genomic sequence has made the mouse indispensable in modeling human diseases and complex syndromes arising from various etiologies. A myriad of approaches have been taken to create mutations in the mouse genome that mimic those in human disorders. Forward genetics mutagenesis projects using various inducers (e.g., ENU, transposons) have been and continue to be executed (Mutagenetix, Australian Phenome Bank, etc. (reviewed in [1]). Many of these screens are designed to look for deviants in one or two specific phenotype areas, such as congenital heart defects or neurobehavioral abnormalities. Once a phenodeviant is identified, mapping or sequencing studies aid in identifying the molecular mutation. More recently, large-scale gene targeted knockout screens have been designed to analyze the phenotypic consequences of mutating each protein-coding gene in mouse (International Mouse Phenotyping Consortium, IMPC) [2]. Unlike previous induced mutation screens, these phenotyping pipelines are designed to systematically screen every mutant mouse line for defects in a wide array of physiological systems. Because the gene mutation is already identified, these phenotype data can be integrated immediately with other information known about the gene’s function, expression and biological pathways.

The Mammalian Phenotype (MP) ontology [3] is a controlled vocabulary that has been used at Mouse Genome Informatics (MGI) to annotate phenotype data from large-scale data sets, including mouse mutagenesis screens, and from data described in published literature. The MP ontology was first developed by iterative additions as curators required terms to describe published and imported phenotype data sets, then later by additions and improvements made via specific review with subject matter experts covering targeted areas of the ontology. Recently, we undertook to add and revise many areas of the ontology simultaneously to accommodate consistent reporting from high-throughput data pipelines and support automated data exchange with the IMPC, MGI and other resources.

## Methods

### Ontology editing and files

The Mammalian Phenotype Ontology in OWL format is maintained and edited using Protégé-4.3 software. Ontology files are available in OWL and converted OBO formats from the MGI ftp site [4].

### Retrieval of MGI data

Data in MGI version 5.20 were retrieved from the public website update posted on 10/21/2014 at MGI [5] or via MouseMine [6].

## Results and discussion

### Expanding and using the mammalian phenotype ontology to annotate high-throughput mouse phenotype data

MP is used as a data standard to annotate published and large scale mouse phenotype data sets [1]. MGI and the Rat Genome Database [7] incorporate this tool to aid in organizing, and analyzing data sets. Unlike other previously imported phenotype data sets to MGI, which required curator intervention to annotate or translate to the MP ontology standard, the high throughput mouse phenotyping pilot projects such as Europhenome [8] and the Sanger Mouse Genetics Project (MGP) [9] are using the MP to annotate data sets directly and the IMPC also has adopted this standard [10]. These large-scale phenotyping projects use a standard series of phenotyping parameters called pipelines (described in detail at IMPC/IMPReSS Pipelines [11]). The IMPC core phenotyping pipeline includes the minimum required phenotype parameters that have been agreed by all IMPC participating research groups. A minimum of seven male and seven female mice at ages of 9–16 weeks are subjected to a battery of mandatory tests with some centers performing added optional tests. Performing these tests and reporting resulting phenotype data in a standardized way allows data to be compared and shared not only among mouse phenotyping centers, but also relative to other annotated published data and contributed data sets.

The accurate description of phenodeviant test results in the IMPReSS pipelines required the addition of 287 new MP terms as of 10/10/2014 (Table 1). New terms were added in multiple systems, with the majority of the new terms (216) assigned in the homeostasis/metabolism section to describe results of specific blood clinical chemistry tests. For example, in Protocol FRUCTOSAMINE IMPC_CBC_020_001 [12] the μmol/l of fructosamine in the blood at 16 weeks of age is measured in one test. This test is used to evaluate the long-term average amount of glucose in blood, and deviations may indicate a problem with regulation of glucose homeostasis. A statistically significant increase is assigned the newly created MP term “increased circulating fructosamine level” [MP:0010087] and a decrease is assigned “decreased circulating fructosamine level” [MP:0010088]. Existing MGI annotations to mutant phenotypes were also updated to use these newly created terms, when appropriate. Other sections of the ontology requiring significant new terms included the immune system, the hematopoietic system and behavior, suggesting that these systems should be subject to further expert review for completeness.

**Table 1:** **MP terms assigned to IMPC parameters, by systems**

**System**	**Terms assigned**	**New terms**
Adipose tissue	6	3
Behavior/neurological	85	15
Cardiovascular system	59	9
Craniofacial	39	1
Digestive/alimentary	6	10
Embryogenesis	3	0
Endocrine/exocrine gland	13	3
Growth/size/body	16	3
Hearing/vestibular/ear	18	3
Hematopoietic system	82	25
Homeostasis/metabolism	216	129
Immune system	67	24
Integument	55	9
Limbs/digits/tail	42	6
Liver/biliary system	1	1
Mortality/aging	10	7
Muscle	5	0
Nervous system	5	4
Pigmentation	13	0
Renal/urinary system	6	2
Reproductive system	25	4
Respiratory system	9	4
Skeleton	70	11
Taste/olfaction	1	0
Vision/eye	55	14

MP terms used in annotations in postnatal tests in IMPC as of 10/10/2014. Note: the total number in the second column is more than 752; this is due to terms assigned to multiple systems, such as “abnormal testis morphology” [MP:0001146], which occurs in both the endocrine/exocrine gland and reproductive systems headings. Some new terms were added during the Europhenome and Sanger Institute’s Mouse Resource Portal pilot phenotyping projects; others were added recently to describe IMPC pipeline parameters.

However, recently reviewed sections of the ontology required fewer additional new terms. For example, the cardiovascular system was recently revised to support the phenotype descriptions of the ENU mutations generated by the Cardiovascular Development Consortium (CvDC) (C. Lo, manuscript submitted). Only 9 additional terms were required to support the IMPC data. Likewise, terms previously requested from members of the FaceBase consortium [13] resulted in good coverage of craniofacial terms, requiring only one new additional term for IMPC in this section.

Many of the new terms created during this revision are now being used in the IMPC tests and in existing MGI mouse phenotype annotations from literature and other resources. MGI phenotype annotations are updated when new terms are added.

Existing ontology structures also were reviewed for content coverage and organization. For example, the term “abnormal adaptive thermogenesis” [MP:0011019] was added as a sibling term to both “abnormal body temperature” [MP:0005535] and “abnormal body temperature homeostasis” [MP:0001777]. “abnormal adaptive thermogenesis” became the parent of the new terms describing stress-induced hyperthermia responses. Recently, new terms covering “abnormal alpha-beta T cell morphology” [MP:0012762] and “abnormal alpha-beta T cell number” [MP:0012763] were added, which organized together the terms describing CD4- and CD8-positive alpha-beta intraepithelial, memory, cytotoxic and regulatory T cells used by the consortium.

### Assignment of MP terms to results of high throughput pipelines

IMPReSS [14] is a database and web portal developed to track phenotyping procedures used by the phenotyping centers of the IMPC. Users can search for phenotype tests such as Lens Opacity [IMPC_EYE_017_001] [15] that assess a phenotype of interest, e.g., cataracts [MP:0001304]. The definition and assignment of these ontology terms is captured in IMPReSS at the level of each parameter and has been developed collaboratively by the data wranglers (scientific support staff charged with assisting centers in data capture and download), the phenotyping centers, and ontology developers. For some parameters, the assignment of phenotype terms by data wranglers of the IMPC was straightforward and did not require further discussion with ontology developers. For example, the significant test results for Heart Weight [IMPC_HWT_001] will be assigned to the MP terms “abnormal heart weight” [MP:0004857], “increased heart weight” [MP:0002833] and “decreased heart weight” [MP:0002834]. For many parameters, a new MP term was requested by data wranglers, but the term assignment was also unambiguous. Examples include many clinical chemistry terms such as “abnormal circulating lipase level” [MP:0011885] and subclasses, “abnormal circulating ferritin level” [MP:0011889] and subclasses or “increased circulating magnesium level” [MP:0010092]. For several terms, clarification of a text definition, or a split of concepts was required. The ontology developer created the new terms “abnormal fluid intake” [MP:0011947], “increased fluid intake” [MP:0011941] and “decreased fluid intake” [MP:0011941] to be used in multiple IMPC parameters, in order to distinguish this phenotype from terms used to describe drinking frequency and other consumption behaviors, for which text definitions were also revised for clarity. Finally, for a subset of parameters, a new term(s) assignment was suggested and created by the ontology developer to describe the results of a test. Such terms include “abnormal bronchoconstrictive response” [MP:0012123] and subclasses, which were recommended for annotation of results in the Enhanced pause (Penh) [ICS_CHL_003_001] plethysmography test that measures response to provocation challenge with antigens/allergens.

752 MP terms have been assigned to protocols in the IMPReSS database as of 10/10/2014, but final assignments/protocols remain under review (Table 1). Existing MGI phenotype annotations were revised to use the newly created terms, when appropriate. However, with some terms, we did not find.

### Use of MP ontology at IMPC

The IMPC web interface at the European Bioinformatics Institute (EBI) [16] allows searching and browsing for phenodeviant data using MP terms. For example, selecting the term “cardiovascular system phenotype” from the phenotypes menu returns a page with the term, definition, all pipeline procedures associated with a cardiovascular system term and all gene variants with cardiovascular system phenotype [17]. Search results may be further refined using available filters. More specific cardiovascular terms, e.g., “abnormal heart weight” can be selected and phenotype data associated with this term may be viewed.

To download and work with large data sets, the phenotype data and MP calls are made available by EBI at the IMPC RESTfulAPI [18]. MP terms associated to the different mutant genotypes may be retrieved in conjunction with the phenotyping center, pipeline, phenotyping procedure, gene symbol, allele symbol, strain name, or any combination of these parameters [2]. MGI uses this interface to retrieve data sets for importation and integration with other MGI data.

### MP expansion to accommodate new IMPC prenatal screens

Identifying genes that are essential during development is required to understand the many processes driving directed prenatal growth, differentiation and organogenesis. Mutations in such genes also can help identify origins of developmental disease and congenital defects. Data currently in MGI suggest that approximately 27% (2669/10014) of genes have at least one knockout allele made into mice that exhibits a prenatal or perinatal lethal phenotype (Table 2).

**Table 2:** **Mouse genes with mutations causing pre- or perinatal lethality**

	**Genes with lethality annotation**	**Alleles with lethality annotation**	**Genes with lethality annotation and postnatal disease annotation**
Prenatal lethality	2017	4393	611
Perinatal lethality	1076	2304	534
Both pre- and perinatal lethality	424	589	322
Total unique objects	2669	6108	823
Ratio of total objects annotated	2669/10014	6697/26894	823/10014

Numbers of mouse genes and alleles involved in genotypes annotated to prenatal or perinatal lethality MP terms in MGI as of 10/21/2014. The first column lists the number of genes with at least one allele in a genotype annotated to a prenatal lethality MP term or a perinatal lethality term. Some genes have multiple alleles annotated to either term set and some genes have one allele annotated to both term sets, possibly due to incomplete penetrance, genetic background differences or the nature of the mutant allele. The second column lists the total number of alleles in a genotype annotated to a prenatal lethality MP term or a perinatal lethality term. The third column lists the number of genes with additional disease annotations suggesting postnatal phenotypes.

To study the large number of homozygous knockout strains generated by the IMPC expected to exhibit a prenatal lethal phenotype, a phenotyping pipeline for the investigation of embryonic lethal knockout lines is being developed. A series of prenatal screenings, lethality staging, gross morphology, and histopathology tests are being discussed by the IMPC to decide upon a logical testing order and to identify additional MP terms specific to these tests [19].

**Figure 1:**
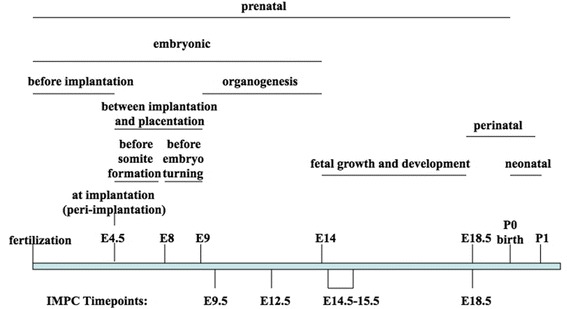
**Mouse prenatal lethality stages.** Defined mouse prenatal stages incorporated in Mammalian Phenotype lethality terms and new time points required to support IMPC prenatal screening (*Not drawn to scale*).

Some tests will require the addition of new MP terms. For example, new early lethality terms may be needed. Existing terms cover windows commonly seen in published literature and can correspond to broad time frames (e.g. “prenatal”) or to narrow time points (e.g. “implantation”) (Figure 1). The IMPC centers collectively have chosen four specific prenatal points for lethality analysis, but not all centers are analyzing each time point. New terms describing “embryonic lethality prior to organogenesis” (approximately mouse E9.5), “embryonic lethality prior to tooth bud stage” (approximately mouse E12-12.5), and “prenatal lethality prior to heart atrial septation” (approximately mouse E14.5-E15.5) have been added and placed in the hierarchy in relationship to the existing terms to cover mouse lines that are not viable at this stage. Additional terms are under discussion. As additional homozygous lethal lines are analyzed, it is possible to identify those that exhibit lethality at E12.5 but viability at E9.5; the window of lethality is somewhere between E9.5 and E12.5. Other centers will only test the E12.5 time point, so a term describing lethality prior to E12.5 may be needed since the E9.5 time point will not be analyzed in this case. There will be more variations of these developmental time windows depending on the testing pipelines finally agreed upon.

The developers of the recently described Drosophila Phenotype Ontology (DPO) [20] have constructed lethality and partial lethality terms for recording and reasoning about the timing of death in populations. The approach taken by the DPO combines the terms “lethal” and “partially lethal - majority die” with a set of terms for life stages from the Drosophila temporal stage ontology using formal semantics in OWL. After reasoning, the resulting list forms a nested classification.

For mouse, there exists defined prenatal stage classifications based on Theiler stages or time from “plug“ after mating, but these as well as postnatal stages are not formalized into a separate comprehensive stage ontology and would be required for considering this approach. Most mouse researchers use embryonic day terminology and not Theiler stages when describing the time of prenatal lethality in mouse in published literature. Further complications to this approach are the significant variations among different mouse inbred strains in their average gestational periods (e.g. 18.75 days in FVB/NJ and 20.5 days in A/J, [21]). Thus the MP uses developmental hallmarks to describe developmental stages, such as “implantation” and “organogenesis”, adding text definitions suggesting an average prenatal age. In addition to the prenatal lethality stage terms, the MP ontology contains lethality terms describing neonatal lethality, early postnatal lethality and lethality at juvenile stages. A temporal stage ontology for mouse using these developmental and postnatal hallmarks would need to be created for such an approach to be feasible for formal definitions within the MP ontology, as well as relating these stages to other species.

To anticipate the need for new MP terms in gross morphology and prenatal histopathology, we are proactively reviewing and adding prenatal MP phenotype terms. New terms covering embryonic pattern formation, gastrulation and organogenesis. We have added over 189 new terms to describe these mutations with greater precision. For example, new terms describing abnormal cardiac or cranial neural crest cell morphology, migration, proliferation, differentiation and apoptosis have been added. Terms describing abnormalities in embryonic neuroepithelium were added. For many other terms, the definitions and synonyms have been updated to include greater detail, including terms describing neural tube defects, neuropore defects and spina bifida.

The embryogenesis section of the MP has been slightly reorganized, with many new and existing terms moved and grouped such as “abnormal gastrulation” [MP:0001695] now placed under “abnormal developmental patterning” [MP:0002084] in the hierarchy, or the new term “abnormal morula morphology” [MP:0012058] placed under “abnormal preimplantation embryo development” [MP:0012103].

In addition to defects of the embryo proper, prenatal lethality may also be due to an indirect result of placental defects. IMPC prenatal screens are also developing tests to distinguish the case in which a placental insufficiency is responsible for lethality. MGI data (retrieved 10/24/2014) includes 356 genes with 593 alleles annotated with terms covering both placental defects and pre -or perinatal lethality. Such mutations may be subject to additional conditional mutation analysis or tetraploid rescue experiments to determine the effects of the mutation on embryonic or adult tissue in absence of placenta defects. We added 27 new placenta related terms to the MP to describe the results of the placenta analysis, for example “placenta necrosis” [MP:0013247].

We will continue to refine and expand the embryogenesis and placenta sections of the ontology, as required for reporting the data generated during the IMPC prenatal phenotype screening.

### Importation of IMPC phenotype data and integration with MGI data sets

The IMPC provides a RESTful interface to mouse alleles, experimental results and genotype–phenotype associations determined by statistical analysis [2]. Phenotyping data were released starting in June, 2014. These data will be retrieved automatically and integrated into all other information in the MGI database. MGI has previously incorporated high-throughput phenotyping data from pilot projects including the EuroPhenome and Sanger Mouse Genetics Project (MGP) pipelines (manuscript in preparation) and new data from the IMPC will be imported similarly. The inclusion of data from IMPC will unify access to mouse phenotype data from many data resources sets and from published data using the Mammalian Phenotype terms as the unifying standard.

**Figure 2:**
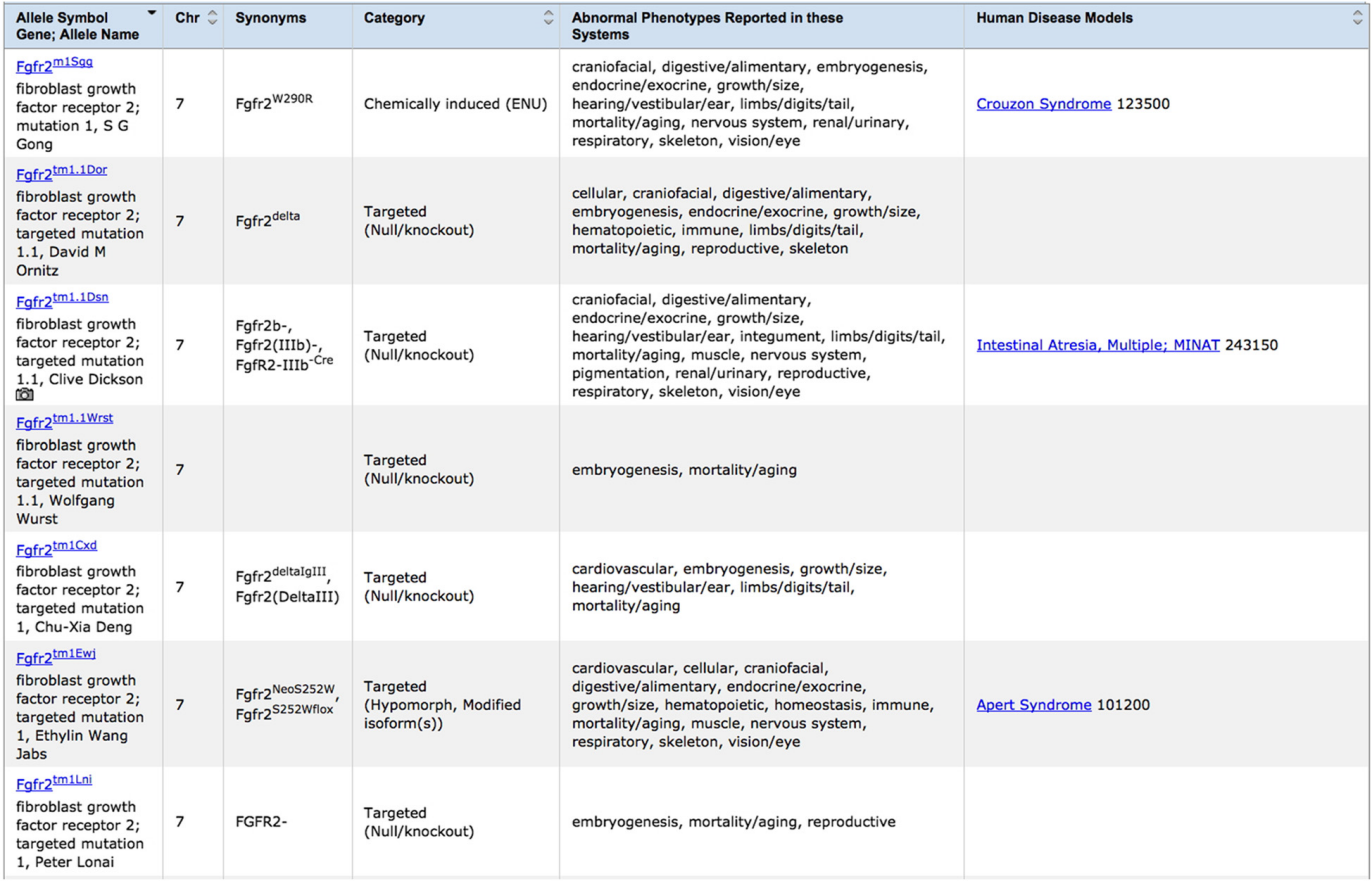
**Allelic series for mouse Fgfr2 gene shows range of phenotypes.** Screenshot of MGI Allele Summary Page listing seven of the twenty-seven known alleles of Fgfr2 that exist in mice. Different mutations in this gene result in a range of phenotypes and disease models in the homozygous and heterozygous states. An additional eighty-eight mutations that exist only in gene trapped or targeted ES cell lines are also known.

MGI will remain the source of global mouse phenotype data integration from large and small scale data sets, contributions and literature. Users will want to see the IMPC knockout data, but also compare these data in context of other types of mutations. Most human diseases are not functional knock-out mutations, so to effectively model human disease, phenotype data associated with all allele types (e.g. induced point mutations (such as ENU), spontaneous mutations (some are recurring), in-dels, copy number variants, conditional mutations, etc.) are required for interspecies comparisons. Of the 3093 genes with an allele annotated to pre- or perinatal lethal phenotypes, MGI data also includes postnatal disease data for 823 of these genes (Table 2). For this set of genes, postnatal annotations involved data from 1) conditional genotypes, 2) haploinsufficient or partially insufficient genotypes when the homozygous knockout is lethal [22], 3) incomplete pre- or perinatal lethality, 4) the influence of mouse genetic background strain which can have dramatic effects on mouse phenotype [23,24], and 5) additional alleles of the gene that were not knockouts, but were small indels, point mutations, etc. that caused altered expression or activity of the gene product (e.g. hypomorphic and gain of function mutations). An example of a gene with an allelic series causing differing phenotypes is Fgfr2 (Figure 2). The *Fgfr2*^*tm1Lni*^ and the *Fgfr2*^*tm1.1Wrst*^ functional targeted knockout mutations result in prenatal lethality. However, the ENU-induced point mutation in *Fgfr2*^*m1Sgg*^ results in a mouse that models Crouzon syndrome. A targeted mutation that introduces a different point mutation, *Fgfr2*^*tm1Ewj*^, results in a mouse that models Apert Syndrome, and a targeted mutation that knocks out only one isoform of Fgfr2, *Fgfr2*^*tm1.1Dsn*^, results in a mouse that models Multiple Intestinal Atresia.

## Conclusions

We describe an expansion of the Mammalian Phenotype Ontology to support phenotype annotation of data generated during high-throughput phenotype screens in mice. Unlike previous phenotyping projects, we have worked with the IMPC and the pilot projects of the Welcome Trust Sanger Institute and Europhenome projects to create and assign phenotype terms to phenodeviants when the data sets are generated by these resources. This will support automated loading of these data from the IMPC to MGI and will also be interoperable with other database resources and tools.

Previously imported small- and mid-scale mutagenesis projects [1] used other system-specific vocabularies to describe phenotypes or used text based phenotype descriptions that required database curator intervention and translation in order to import the phenotype data into MGI using the Mammalian Phenotype Ontology standard. The IMPC data will be loaded directly into MGI and integrated immediately with all other allele and data types to support knowledge discovery. Furthermore, the MP also is used by mouse repositories to enable searching and describing available mouse strains and stocks that were originally generated for the high throughput phenotyping screens. These include the Jackson Laboratory Repository [25], the European Mouse Mutant Archive [26], the Mutant Mouse Regional Resource Centers [27], and the KOMP Repository [28] among others.

## Abbreviations

IMPC: International Mouse Phenotyping Consortium
MP: Mammalian phenotype
MGI: Mouse genome informatics
OWL: Ontology web language
OBO: Open biomedical ontologies
RGD: Rat genome database
MGP: Mouse Genetics Project, JAXMice, Jackson Laboratory Repository
IMPreSS: International Mouse Phenotyping Resource of Standardised Screens
EMMA: European mouse mutant archive
MMRRC: Mutant Mouse Regional Resource Centers, KOMP, Knockout Mouse Repository
